# Treatment of Inflammation

**Published:** 1864-09

**Authors:** John Hughes Bennett


### ART. II.—*Treatment of Inflammation.* By JOHN HUGHES BENNETT, M. D., F. R. S. C., &c.

Formerly it was supposed that the essential phenomenon of inflammation consisted in the alteration in the blood and blood vessels. The views previously detailed seek to establish that this process really consists in irritation of the extra-vascula elements of the textures, producing exudation of the liquor sanguinis. The former doctrine naturally led its upholders to maintain an antiphlogistic treatment; the latter one has naturally led to an opposite practice. There is no inflammation so well capable of testing the value of any particular treatment as a pneumonia: first, because there is none that can be determined by functional symptoms and physical signs; secondly, because the perturbation of the system and importance of the organs involved have ever, and must always, attract strongly the attention of medical men; thirdly, because it, perhaps, more than any other, has been supposed to be amenable to bloodletting and antiphlogistics. It is now eighteen years since a careful investigation into the pathology of inflammation induced me to doubt the value of the then universal practice in these cases, and this for the following reasons:

In the first place, the cause of the inflammation is an initiation of the textures—of the ultimate molecules of the part, in consequence of which their vital power of selection is destroyed, and that of their attraction is increased. The removal of blood by venesection cannot alter this state of matters—neither can other lowering remedies. If the inflammation be superficial and limited, local bleeding may relieve the congestion, as in conjunctivitis, but if exudation has occurred it cannot remove that.

In the second place, an exudation of true inflammation having occurred, it can only be absorbed by undergoing cell transformation. Now, this demands vital force or strength, and is arrested by weakness. Hence, inflammations in healthy men rapidly go through their natural course; in weak persons this is delayed; hence their fatality.

In the third place, the strong pulse, fever, and increased flow of blood in the neighborhood of inflamed parts have been wrongly interpreted by practitioners. They are the results, and not the causes of inflammation, and show that the economy is actively at work repairing the injury. So far, therefore, from being interfered with and interrupted they should be encouraged—locally by warmth, which also relieves pain, and internally by nutrients.

It follows, fourthly, that if these views be correct, our object in the treatment of inflammation should be directed towards bringing the disease to a favorable conclusion by supporting rather than diminishing the vital strength of the economy, and this not by over-stimulating, as was done by Dr. Todd, but simply by attending to all those circumstances which restore the nutritive processes to a healthy condition.

Having been guided by these views in my practice for the last fourteen years, and having seen that gradually they have been adopted by the profession, it is, I think, in my power to offer you the most convincing proof of their correctness by contrasting the results of an antiphlogistic treatment, as formerly practiced in pneumonia, with those furnished by an analysis of 105 consecutive cases that have been carefully recorded by my various clinical clerks in the Royal Infirmary.

With regard to a past antiphlogistic practice, there can be no doubt that pneumonia, during the period it was treated by bleeding, antimonials, and other antiphlogistics, can be proved to have been a very fatal malady. Andral tells us that the experience of ages has taught us to be more prodigal in the taking of blood in pneumonia than in any other disease; that there is no period of the disease, no condition of the pulse, no apparent debility of system, no age, which forbids its practice. Yet it is curious to observe that of the 65 cases of the disease he records in the "Clinical Medicine" illustrative of his treatment, no less than 36, or more than a half, die. Of the uncomplicated cases, 9 only reach the stage of engorgement, yet 2 of them die; 13 reach the second stage, and of these 5 die; 7 cases reach the third stage, and of these all die. Of the 29 uncomplicated cases, 14—nearly one half—die. He gives 36 complicated cases, and of these 22—nearly two-thirds—die.

The facts recorded by the physicians of the Edinburg Royal Infirmary between the years 1832 and 1837, as tabulated by my former resident clerk, Dr. Thorburne, give a mortality of 1 death in 3 cases. The statistics of Dr. John Reid, in the same institution, between the years 1839 and 1849, give the same mortality of 1 death in every 3 cases of pneumonia. The numbers are—cases, 648; deaths, 222. The carefully chosen cases of M. Louis, to test the effects of bloodletting, give exactly the same results: the cases were 107; deaths, 22.

Rasori thought it a great improvement in practice when, by means of his antimonial treatment, he reduced the mortality in cases of pneumonia from one in three to one in four and a half—that is, in 648 cases, 143 died. Grisolle, on diminishing the amount of bleeding, still further reduced the mortality to one in six and a third; and Dietl, by a purely expectant treatment, brought it down to one in thirteen.

My practice is directed to support the strength of the economy; never to weaken it in any stage by antiphlogistics, although, if dyspnœa be urgent, cupping, or a small bleeding, may be practiced as a palliative, more especially in bronchial or cardial complication. During the febrile excitement, mild salines are administered. On the fourth or fifth day, when the fever abates, good beef tea and nutrients are given, and on the pulse becoming soft or weak, from four to eight ounces of wine daily. As the period of crisis approaches, slight diuretics are given to favor the excretory process.

The results of this practice in 105 cases of pneumonia in adults, consecutively treated by me in the clinical wards of the Royal Infirmary, during the last fourteen years, are as follows:

Number of cases, 105; deaths, 3; all complicated cases—one of intestinal ulceration, one of Bright's disease, and one (a drunkard) with delirium tremens and cerebral meningitis. Ratio of deaths, one in thirty-five cases. Average age of cases, thirty-one and two thirds years.

| | | | | | |
|---|---|---|---|---|---|
| Single uncomplicated cases | 58 | duration averaged | | 13½ | days |
| Double " " | 19 | " | " | 20 | " |
| Complicated " | 17 | " | " | 15 4-5 | " |
| Unsatisfactory cases as to duration | 8 | | | | |
| Deaths | 3 | | | | |
| | 105* | | | | |

Average residence in hospital of 77 uncomplicated cases of pneumonia (single and double) was twenty-two and an eighth days. (This is too high; some linger from weakness, from subsequent attacks of rheumatism, or skin disease. One remained in a fortnight after recovery, from having no shoes, &c.)

It has been supposed from this comparatively small number of cases, ranging over so long a period as fourteen years, the disease is rare in Edinburg; but it should be explained that the clinical professors are on duty alternately, and as regards myself, I have never acted as physician to the infirmary more than one half the the year, and in most cases only one-third of the year. Again, it has been supposed from the small mortality that the cases there are unusually slight and trivial, or that the disease is not extensive. But it is not so. In Edinburg now, as formerly, many, and especially the double cases of pneumonia have been very severe, with great dyspnœa and very urgent symptoms. I have also frequently pointed out instances of the pulse being hard and strong in vigorous young men, in whom, however, the most rapid recoveries were invariably observed. It should also be noted that these cases were in no way selected, but do not include a few who were admitted *in extremis* at night, and never seen by the physician, nor such as were partly treated by the physicians in the hospital, and for which treatment I am not responsible.

From these facts I conclude—

1st. That simple pneumonia, if treated so as to support instead of lower the nutritive processes, so far from being a fatal disease, almost invariably recovers.

2d. That the cause of mortality in these cases is exhaustion, either before they come under medical supervision, or, as formerly practiced, from an antiphlogistic or a lowering treatment. All bleedings that do not exhaust must be regarded as palliative rather than as curative, and their influence has yet to be determined with exactitude.

3d. That the same rule applies to all inflammations, the amount of danger being in direct ratio to the weakness of the system and the existence of complications in the disease, especially blood-poisoning.

I cannot dwell at greater length now on what it appears to me are these important results. I shall only remark, in conclusion, that in my opinion they are not the effect of chance; of empirica experiment; of a change in the nature of inflammation, or of the force of the pulse in man and animals; of an alteration in diet or of drink, or of nervous susceptibility; nor of a change in the type of disease; all of which have been supposed by some to be explanatory of facts which can no longer be denied. The more I consider this subject the more am I convinced that it is to the advance of medical science only that it can be rightly attributed, and that it is our highest privilege and honor so to consider it. Indeed, no stronger proof can be offered of the improvement in practice that has resulted from a more correct pathology, than the diminished mortality and great success which it has been shown now attend our treatment of acute inflammation.

* Since these statistics were made up, I have had ten other cases of pneumonia, all of which recovered; making the total number 115.

